# The emergence of collective knowledge and cumulative culture in animals, humans and machines

**DOI:** 10.1098/rstb.2020.0306

**Published:** 2022-01-31

**Authors:** Andrew Whiten, Dora Biro, Nicolas Bredeche, Ellen C. Garland, Simon Kirby

**Affiliations:** ^1^ Centre for Social Learning and Cognitive Evolution, School of Psychology and Neuroscience, Scottish Oceans Institute, School of Biology, University of St Andrews, St Andrews, UK; ^2^ Centre for Social Learning and Cognitive Evolution, and Sea Mammal Research Unit, Scottish Oceans Institute, School of Biology, University of St Andrews, St Andrews, UK; ^3^ Department of Zoology, University of Oxford, Oxford, UK; ^4^ Department of Brain and Cognitive Sciences, University of Rochester, Rochester, NY, USA; ^5^ Sorbonne Université, CNRS, Institut des Systèmes Intelligents et de Robotique, ISIR, 75005 Paris, France; ^6^ Centre for Language Evolution, University of Edinburgh, Edinburgh, UK

**Keywords:** collective cognition, collective memory, social learning, culture, cultural evolution, cumulative culture

## 1. Introduction

The goal of this themed issue and the associated Royal Society and British Academy joint Discussion Meeting is to advance, and bridge between, two topics and their respective research fields that have burgeoned in recent years, although to date they have often done so quite separately. One field is concerned with collective action, collective intelligence and collective knowledge among groupings of individuals; phenomena in which significantly more is achieved by the collective than is possible for any one individual alone. Their manifestations and mechanisms have been studied across populations of the kinds of non-human animals (henceforth ‘animals’), humans and machines that are the subject matter of this issue. These have been flagged by a variety of expressions across animal and human studies including, for example, ‘consensus decision-making’ [1], ‘the wisdom of the hive’ [2], ‘quorum decision-making’ [3], ‘emergent sensing’ [4], ‘collective intelligence’ [5], ‘the wisdom of the crowd’ [6], ‘collective brain’ [7], ‘group cognition’ and ‘extended mind’ [8,9], ‘group-mindedness’ and ‘collective intentionality’ [10]. In artificial intelligence research there is ‘swarm robotics’ and ‘collective robotics’ [11–17]. For Mulgan [18], ‘Big Mind’ includes both the latter two domains, integrated in the collective intelligence generated through large-scale human–machine interactions. Few attempts have been made to integrate the contributions reflected in this diversity of terminologies. In this issue, we address the emergence and evolution of these phenomena, appraising commonalities and differences among them.

The second field of interest covers the emergence and evolution of the collective entity we call *culture*—the creation, transmission and spread of traditions through social learning (learning from others), in humans, animals and machines. Culture has long been presented as a unique defining feature of humans [19,20]. It pervades virtually every aspect of what it is to be human [21,22]. Yet, recent decades have revealed that culture, defined as above, plays a significant role in the lives of numerous vertebrate taxa and perhaps of invertebrates (notably insects) too [23–27]. The study of social learning in robots, reviewed in this issue [28–30] also raises the prospect of cultural evolution in the world of machines.

The research literatures focused, respectively, on collective knowledge and culture (including each of the animal, human and machine research streams within them) have been built largely as separate endeavours. In this issue, we seek not only to extend them but also to examine emerging links between them. Exploring such connections, Biro *et al*. [31] pointed out a largely overlooked aspect of collective animal behaviour: that many of the collective outcomes that have been identified may be contingent on the collective's previous history and collective memory. Collective knowledge may change over time when the same group members solve the same task repeatedly, or across partial turnovers in group membership, amounting to the emergence of a group culture. This may in turn extend to cultural transmission of collective knowledge across generations. Any additional extensions in such collective cultural knowledge may in turn evidence cumulative culture.

An example of such processes in action was provided by an experiment with homing pigeons [32]. In this study, two pigeons were first repeatedly tracked as they made homing flights. Then, one of the pair was replaced by a naive bird and the new combination again flew repeated flights, before the most experienced was again replaced by a naive bird. Later pairs of pigeons in these transitions were thus different to those flying earlier in the sequence, yet homing flight paths became increasingly efficient and direct over time. The experiment accordingly demonstrated (i) the transmission, across pairings, of information underlying good flight paths generated up to that point; (ii) a capacity of consecutive pairs not only to share this information, but in interaction, improve it; and thence (iii) create cumulative cultural progress across the whole sequence of repeated replacement pairings.

How might such processes play out in nature? Long-term records of wild bighorn sheep suggest one answer [33]. In the United States, groups of these animals were translocated, sometimes as much as two centuries ago, to areas in which the species had been extirpated. Here, in territory unknown to them, they failed to exhibit the annual ‘green wave surfing’ in which these animals normally pursue a series of higher and higher altitude springtime patches of graze. However, this behaviour re-emerged in the translocated populations, and the knowledge and skill underlying the ability to optimize foraging movements in time and space was found to grow progressively over decades and over generations. The authors concluded ‘that ungulates accumulate knowledge of local phenological patterns over time via the ‘ratcheting effect’ wherein each generation augments culturally transmitted information with information gained from their own experience, a process known as cumulative cultural evolution’ ([33, p. 1024], citing [34]).

## 2. Core research questions

Such emerging links between the topics of collective behaviour, knowledge sharing, collective knowledge generation and culture, and a range of other relevant advances in studies of animals, humans and machines lead us to pursue a set of core questions as follows:

— how do collectives, whether of animals, humans, robots or other machines achieve shared levels of knowledge/information, beyond those accessible to constituent individuals acting alone?
— in what ways and under what conditions are these processes extended temporally, such that collective contributions are maintained and persist over significant time depths?
— to what extent and in what ways do these kinds of collectives create new behaviours or artefacts when they combine and integrate different contributions from different individuals, and do these spread via social learning? and
— in what ways may these phenomena recur across multiple generations of cultural transmission, creating the potential for extended cumulative cultural transmission?

Of course, many more specific but related questions are addressed in the articles assembled in this issue. We do not provide extended summaries of these articles here. Prospective readers can consult the abstracts. Instead, we outline the key background literature and briefly indicate how the articles in the issue relate to and advance these.

## 3. Collective behaviour, knowledge and culture among non-human animals

Much of the animal literature to date focuses either on collective phenomena per se, or on culture-related phenomena, and accordingly we introduce these separately here.

### (a) Collective behaviour and knowledge in animals

An extensive literature has accumulated, particularly this century, analysing collective actions in animals. Contexts studied include the collective movements of large aggregates (bird flocks, fish schools, insect swarms and ungulate herds [35–37]), collective construction (foraging trail networks, social insect nests and even structures composed of the bodies of the animals themselves, such as ant-bridges [38–40]), the timing and coordination of collective activities (the emergence of synchrony in firefly flashing and in predator evasion [41,42]) and collective decision-making in the group's choice of when, where and what actions to implement [43]. Key principles identified emphasize that much of collective animal behaviour is ‘self-organized’ [44], relying on relatively simple local interactions of individuals with their neighbours and with the environment, in the absence of any ‘global’ overseer or leader. Complex, robust and scalable group-level phenomena emerge that are not necessarily predictable from observing any single individual by itself.

In this issue, we are additionally focused on contexts in which *information* is linked collectively in ways that evoke such terms as ‘collective memory’ [45], ‘collective decision making’ and ‘collective minds’ [46]. The justification for such references to concepts of cognition and knowledge, even where the cognitive powers of individual animals or robots may be relatively elementary, can be illustrated in an analysis of chemical communication among ants [47]. Ants mark trails with pheromones of both low and high volatility, allowing collective route maps to be accumulated and refined over time. This provides both long-term memories of potentially useful foraging routes, and short-term memories coding currently valuable routes to resources. The latter ‘working memory’ facilitates the ‘attention’ of the colony as a whole, adaptively guiding the workforce's learning and exploitation as resource distributions change.

Collective decision-making has been extensively studied in the context of negotiated route choices, in situations where component individuals initially express different preferences based on their personal knowledge to date. For example, despite living in societies heavily structured by dominance relationships, at a fine spatio-temporal scale baboons are more likely to follow routes suggested by multiple individuals than to follow the most dominant [48]. When directions suggested in the actions of initiators differ by only relatively small angles, the group typically follows a compromise direction between them, but if the angle is large, just one is chosen. The latter choice tends to occur as a majority of the group progressively express a preference for it. The pattern of ‘compromise’ decisions being made when the angle of disagreement is small, and a ‘winner takes all’ decision emerging when it is large, has been found in a variety of analogous contexts and species, including the choice of homing paths in pigeons [49] and of travel directions in wild vulturine guineafowl [50]. Similarly, the principle of the numerical majority dictating the group consensus is a well-known effect characterizing the selection of new nest sites by colonies of honeybees and ants [51,52], and has also been shown in human crowds instructed to follow a set of simple behavioural rules simulating those underlying collective animal movement [53].

In these cases, individuals often vary in their knowledge of a single factor, such as the location of a resource like fruiting trees or nest-sites. However, knowledge concerning different factors might be in play even in contexts like those outlined above: for example, some baboons may try to initiate movement towards a food resource they know of, while others initiate alternative routes to avoid an area they know has recently been frequented by a significant predator. Some individuals may know about only one of these, yet the group as a whole may be able to generate an adaptive overall response that weights both factors. Indeed, the specific weighting that different individuals' inputs receive may vary according to their current physiological needs, motivational states, or the reliability of their information, leading to more flexible integration of pooled preferences [54]. For example, in plains zebras, lactating females—i.e. those with the highest water and energy needs—are more likely to influence the herd's movements than non-lactating ones [55].

Alternatively, collective responses can also be the cumulative output of individual inputs rather than their (weighted) average. The potential complexities of such additive effects are challenging to tease apart in nature, but have been explored in controlled experimental contexts. For example, Webster *et al*. [56] tested sticklebacks faced with a two-step task in which the first step was navigating through a structured environment to a food patch, and the second step was accessing the food by swimming through a small hole. Some shoals contained only entirely naive fish, whereas others mixed naive fish with fish already experienced in the navigation step only, or experienced in the food access step only. In a fourth combination, the group included equal numbers of naive fish, and others trained in either navigation-only or food access-only. Results clearly showed that these mixtures were superior to the others in the proportion of the group successfully entering the feeder and the speed with which they were able to do so, demonstrating what the authors describe as adaptive ‘experience pooling’.

Although not tracked in that study, from what we know of social learning in fishes [57], all the fish whose discovery of this foraging innovation was enhanced by the experience pooling would probably be more successful at the task subsequently, implicating a form of social transmission. Such effects illustrate one potential link between collective knowledge, social learning and culture, when a perspective with some time-depth is taken. Outstanding key questions concern the role of the collective (its size, composition, stability and life history) in generating new information not discoverable by single individuals, the role of individual heterogeneity in where and how much of this information is stored as the group's ‘collective memory’, the time-depth of the latter, and context-dependent effects on the retrieval and execution of appropriate behavioural solutions in collectively learnt tasks.

### (b) Social learning and culture in animals

Culture was defined above as the creation and spread of traditions through social learning (learning from others). Traditions can in turn be defined as behaviours, artefacts or other entities that are shared within a community and maintained over significant periods, including across generations. Once thought unique to humans, over the last 70 years or so, culture conceptualized in this way has been discovered to play a significant part in the lives of an ever-expanding range of animal taxa including a variety of mammals, birds, reptiles and fishes [23–27]. Such evidence has emerged in some cases through the accumulation of long-term field studies, but also through the refinement of a diverse portfolio of methodologies, from controlled experiments in both laboratory and field, to statistical techniques to identify the signature of cultural transmission in the ways that innovations spread preferentially through social networks [58]. Here it is important to recognize the distinction between evidence for social learning per se, and that required to identify cultural transmission, through the demonstration of repeated episodes of social learning as innovations diffuse within and/or across groups and generations. Much of what animals learn socially may have only transient significance, such as the location of a short-lived resource, and never become the basis for a tradition. The identification of social learning of a preference for particular flowers by bumblebees [59], for example, thus does not in itself establish culture. However, laboratory-based experiments have gone on to demonstrate cultural transmission along chains of learners, including in the foraging behaviour of bumblebees [60] and mate-choice copying of fruit flies [61]. What remains unclear is the reach of culture in insects' lives in the wild.

In vertebrates, the diversity of behavioural repertoires found to be shaped by cultural transmission has progressively expanded alongside its taxonomic diversity. Examples include foraging techniques [62,63], tool use [64,65], vocal communication [66,67], social customs [68,69], preferences for particular prey [70] and other dietary items [71], migratory pathways and destinations [72,73] and mate characteristics [61].

Cultural transmission spans not only this diversity of functions but may also pervade successive phases of animals’ life history. In species with parental care, much appears to be transmitted during this initial phase of dependence, but in group-living animals ‘horizontal’ transmission between peers and ‘oblique’ transmission from non-parental adults can be important at later ages, including a renewed role for learning from others as adults disperse to new groups and ranges unfamiliar to them [71,74]. A recent book-length review of the accumulating evidence for cetaceans concluded that culture so permeates whales' lives that ‘culture is a major part of what the whales are’ [23, p. 17]. In similar vein, Schuppli & van Schaik [75] argue that prior approaches to identifying the scope of ape cultures that rested on comparing different communities have much underestimated the role of culture in these animals' lives, neglecting local ecological adaptations and cultural universals. By instead logging all the contexts in which juveniles closely peered at adult behaviour patterns, a measure the authors earlier validated as indexing social learning, studies of two orangutan populations indicated as many as 125 and 190 culturally transmitted behaviours, respectively. The authors concluded ‘that immatures learn virtually all of their skills socially’ [75, p. 5].

Culture may pervade many animals' lives, but does it evolve, as human culture so manifestly does? The answer depends in part on what counts as evolution. If, as can be the case in evolutionary biology, we are talking of only cultural *change*, then there is growing evidence for the occurrence of cultural evolution among animals, as long-term studies accumulate [25,27]. Changes in birdsong logged over periods that have now extended over several decades have provided the greatest number of peer-reviewed publications that index ‘cultural evolution’ in their titles [25]. Long-term changes were also identified in the spread of a ‘lob-tail’ technique of predation in humpback whales over 26 years [63], but some changes can be much faster, as when a new form of sponge-making to aid drinking emerged and spread in chimpanzees over only approximately a single week [76].

Human scientific lives are short compared to many of the potential evolutionary changes (whether based on genetic or cultural inheritance) that interest us. Archaeology has begun to change this somewhat for animals, as it has for human cultural change. Archaeological excavations focused on the types of stones used to crack nuts by modern-day bearded capuchins pointed to four different evolutionary phases spanning 3000 years, marked by changes in size, wear, percussive battering and anvil types [77].

A more demanding concept of cultural evolution requires it to be cumulative, such that ‘some measure of performance’ is progressively enhanced [78, p. 2]. In our introduction, we already noted two quite different examples of this, an experimental study of pigeon homing [32] and an analysis of long-term records of green wave surfing skill in bighorn sheep [33]. Both of these concern enhancements in movement efficiency, but other examples have begun to extend the diversity of species and behavioural arenas in which such cumulative cultural change has been identified. One such study showed cumulative cultural evolution (CCE) in the capacity of baboons to recognize and transmit visual patterns, in an analogue of similar changes in the cultural evolution of artificial languages in human experiments [79,80]. Another study tracked changes in the songs of humpback whales over 12 years, during which periods of rising complexity alternated with falls occurring when radically new songs (cultural revolutions) emerged [81]. All such cases are suggestive of collective contributions of different animals' innovations to cumulative cultural change, across significant time-depths. That of the humpback whales is pursued in greater depth in this issue [82].

## 4. Collective knowledge and cumulative culture in humans

Studying the wisdom of collectives is often traced back to Galton [83], who showed that the sometimes widely differing estimates of the weight of an ox at a county fair, when averaged, came surprisingly to within 1% of the true weight. Since that time, studies of a variety of related collective cognitive phenomena have generated large and growing literatures in fields as varied as economics, psychology, sociology, law, political science and anthropology [84]. However, ‘culture’ was not even indexed in the latter ‘*Handbook of collective intelligence*’.

Similarly, collective knowledge/intelligence was not in the index of the most recent wide-ranging compilation of research findings from the field of human cultural evolution [85]. The study of cultural evolution can be traced at least as far back as works by Schleicher [86] concerning language evolution, well known to Darwin [87]. With time, evolutionary analyses were extended to fields such as weaponry and other technologies [88]. The modern field of cultural evolution is generally traced to foundational systematic analyses by Cavalli-Sforza & Feldman [89] and Boyd & Richerson [90], its maturation marked in 2017 by the first international conference of a ‘Cultural Evolution Society’. A growing number of studies have, however, begun to integrate the topic of collective knowledge with that of culture [91]. Here, we introduce examples that offer foundations for articles in this issue that carry this work forwards.

### (a) Inferences about collective information and culture in past and present foraging societies

Two very different empirical sources, both represented in this issue, focus particularly on reconstructing our evolutionary past. There is an important complementarity between the two given the strengths and weaknesses of each. One is the archaeological record, including fossils, genomic material and particularly artefacts dating from the stone age to the present day [92]. The artefacts provide us with a progressively more detailed record of the evolution of human material culture, but limited inferences can be drawn about social matters such as collective action and knowledge. By contrast, rich data on the latter come from the study of present-day peoples dependent on the hunting and gathering (HG) ways of life that the archaeological material tells us characterized millennia of our species' recent history [93]. However, the cultures of these communities tend to be very stable, revealing little of evolutionary change, whereas change permeates the archaeological record of the last few million years. The two perspectives thus enrich and inform each other in multiple ways.

Studies of Hadza HG in Africa and Ache HG in South America suggest that the collective cultural knowledge of these peoples is structured in significant ways by forms of social interaction that differ much from those of great apes [94]. These include intermittent and friendly interactions between bands. Estimates from quantitative studies of these peoples suggest that across their lifetimes, Hadza and Ache men are likely to be able to observe as many as 300 men engaged in tool making across different bands. This contrasts strikingly with the lives of male chimpanzees, whose inter-community relationships are extremely intolerant and typically marked by lethal raiding. Hill *et al*. [94] suggested that the scale of information exchange observed in HG societies is likely to have been a key factor in facilitating cultural transmission and particularly the scope for CCE to take place.

Parallel conclusions have been derived from archaeologically based analyses identifying correlations between reconstructed demographic changes and major transitions in material culture in late Pleistocene times [95]. Population densities and migration patterns inferred from genomic data were shown to attain levels predicted to promote marked acceleration in cumulative cultural change at two significant spatio-temporal junctures. The more recent, at approximately 45 ka in Europe and western Asia, represents the Upper Palaeolithic transition marked by an extensive cluster of cultural advances including fine stone tools and sophisticated weaponry like spear throwers, together with realistic art and body decoration materials. The earlier juncture, around approximately 100 ka, corresponds to what are thought to be the first archeological signatures of such cultural advances in Africa.

Of course these scenarios concern a much larger-scale inferred linkage between the promotion of collective knowledge through major demographic shifts and cultural advances, than the recent studies of HG populations. They also rely on a significant number of assumptions and inferential leaps, given the fragmentary nature of archaeological evidence bases. Studies of contemporary HG life, therefore, provide invaluable and complementary concrete observations of the collective and cultural phenomena of interest.

For example, a study of the forms of usage of 33 plant species by 219 BaYaka HG individuals in Congo revealed a hierarchical structure in their collective knowledge [96]. Knowledge of medicinal usages are principally shared at the level of the family—between spouses and both biological and affinal kin, whereas collective knowledge concerning social norms such as ritual usage, and foraging requirements, occurs at the level of the camp. The authors proposed that multi-family camps provide a framework facilitating the exchange of social and functional information and ultimately potentials for cumulative cultural change.

Wireless sensing technology, in which small ‘mote’ devices worn by individuals provide objective information on social networks across large populations, have now been used to delineate such multiple-level HG social structures in detail [97]. For the 53 adults in seven Agta camps in forests in the Philippines, 59% of all the possible close dyadic interactions were recorded as occurring within a band in a single month, plus an additional 28% of the possible dyadic interactions between camps. For 37 adults in three coastal camps, the equivalent figures were 85% and 56%.

A number of studies converge to suggest that such hierarchical structuring may facilitate CCE because of the mix of fragmented collective knowledge arising across camps, coupled with intermittent exchange between them. Gorillas have been recorded to use nine of the medicinal plants used by the BaYaka studied by Salali *et al.* [96], and chimpanzees six, whereas the BaYaka use 32 [96]; but significantly, the study found that no one individual knew all of them. Laboratory experiments and computational modelling studies have suggested what may be the significance of this [98,99]. These latter studies found that where a population is segmented into groups (as occurs in the HG camps in the studies above), CCE is facilitated when ties across the whole social network are partial, rather than fully connected (where every individual can socially learn a new innovation from any other). This is because partial connectivity encourages diversity in solutions to problems faced, which can be combined to achieve advances in ways that do not occur in a more completely connected network. Modelling of alternative networks suggests that an intermediate level of partial connections can be optimal, with too many direct learning connections inhibiting solution diversity, and too few failing to generate combinatorial advances [99].

In natural groups, such as those of HGs, such processes may occur over such long timeframes that they are difficult to address in scientific lifetimes. Accordingly, Migliano *et al*. [100] instead ran a version of the laboratory experiment of Derex *et al*. [98], simulating the results of inserting the real life demographies they delineated as described above. Results showed that the multi-level structuring of this HG society, including families within camps and multi-camp clusterings, did indeed generate high levels of CCE, in comparison to more fully connected networks of the same size.

Genomic analyses identifying relatedness of individuals in shared burials dating to 35 ka have concluded these match those among contemporary HGs described above [101], suggesting that the associated patterns of collective knowledge and potentials for cumulative culture have an ancient evolutionary history. There is further evidence that hominins were transporting cultural materials including ochre from iron-rich rocks and materials for manufacturing stone tools over distances of up to 50 km as long as 300 ka [102]. There are surely rich prospects for further cross fertilization and integration of related findings from network science, archaeology, paleodemography and cultural evolution [103].

### (b) Laboratory experiments tease apart causal linkages between collective cognition and culture

Laboratory experiments offer much greater power to identify and dissect causal pathways. A weakness can be their ecological validity, but each methodology has its strengths and weaknesses. Experiments involving ‘micro-societies’ in the laboratory complement the real-world approaches outlined above and create further potential for cross fertilization, one example of which we have already noted.

Caldwell & Millen [104] pioneered such experiments, creating transmission chains in which individuals were successively added to and lost from micro-societies of four individuals repeatedly faced with a task such as building spaghetti and plasticene towers ever higher. These demonstrated cumulative cultural progress on such tasks, amounting to an expression of collective intelligence spread across the successive cultural ‘generations’ of the experiment.

Building on this work, Muthukrishna *et al*. [105] contrasted the effects of transmission chains in which in each generation, one naive individual could learn from just one other, versus chains where in each generation five naive individuals could observe and learn from five others. Using more complex tasks than the spaghetti tower building, arguably reflecting those occurring in our species past, such as complex knot tying, this study showed markedly greater cumulative success in the condition with multiple models available; indeed after 10 cultural generations, all individuals in the last phase of the five-model condition exhibited more skill than the most skilled in the one-model condition. Kempe & Mesoudi [106] obtained similar results with a different task, contrasting a one-individual condition with a three-individual condition.

Further analysis in the Muthukrishna *et al*. study showed that in the multiple-models condition, individuals were influenced most by the most skilled person they could observe, but they also relied to some extent on the best four of the five available. The authors speculated that ‘by drawing ideas, techniques and insights from different models, learners can end up with novel re-combinations that none of their cultural parents possesses. This, in a sense, creates innovations without ‘invention’, ‘creativity’ or trial and error learning’ ([105, p. 5]; see also [107]). In other variants of these micro-society experiments, researchers have further explored the decision-making involved as well as the role of group size, finding that outcomes are influenced through complex interplays with reasoning abilities [108], pay-offs of self's versus others’ solutions, conformity and similarities between self's and others approaches [109].

These studies began by testing and finding evidence favouring theories that greater group size facilitates cultural evolution. Further work, however, has provided evidence that this is an over-simplification, because the strongest effects occur in population networks composed of sub-groups in separate neighbourhoods, while being partially or intermittently connected [98,99,108–110]. As noted in the section above, these findings concur well with those arising from recent studies among real-world HG social networks [93,94,96,97,100].

Laboratory transmission chain experiments have become important in evolutionary linguistics [111] where they are used alongside computational models [112] to explore how cognitive biases in learning and communication shape linguistic structure. The techniques extend the artificial language learning method [113] from psycholinguistics to examine what happens when, instead of learning a miniature language provided by the experimenter, participants instead learn from the output of a previous participant in the experiment—a process called iterated learning [114]. More recently, iterated learning has been extended to include interaction between participants who use the emerging language to solve communication games [80].

This work suggests that many of the universal design features of language are the product of populations optimizing a trade-off between two pressures on language: a pressure to be simplified to facilitate learning; and a pressure to be informative, and therefore useful for communication [80,115]. Cultural evolution is the adaptive process that delivers this optimization. With this realization, evolutionary linguists have started to examine differences in language structure between different types of populations—a topic that had been taboo for many years owing to assumptions of uniformitarianism [116]. For example, Lupyan & Dale [117] present evidence that languages spoken by a larger number of speakers are structurally simpler than ones spoken by smaller populations. This suggests a new research programme for evolutionary linguistics in which iterated learning and communication experiments are run in populations with different numbers of individuals to see which types of population, both in terms of size, but also social network structure more generally, lead to simpler or more complex language structures [118,119].

### (c) Developmental studies

For many of the studies outlined above, ‘emergence’ of the phenomena of interest is framed in evolutionary and phylogenetic terms, but emergence also occurs in the course of development. Synthesizing the findings of two decades of intensive research on the socio-cognitive development of children, in many cases directly contrasted with that of chimpanzees and other apes, Tomasello [10] discerns two major milestones, the second of which he labels ‘collective intentionality’, occurring around 3 years of age. This is built on the foundations provided by the earlier milestone, ‘joint intentionality’, typically emerging around nine months of age. This is characterized by episodes in which coordination is achieved between infants and others in jointly attending to and cognitively engaging with external entities, such as pointing to target objects and beginning to mark them in other ways, such as through vocalizations and holding up items of interest.

The later phase of collective intentionality is additionally characterized by child and interactant communicating, in the most explicit instances verbally, in ways that indicate the child appreciates the two may hold different mental perspectives. Thus, there is ‘joint attention to mental content’ in this micro-collective. The child is here described as conceptualizing a group-minded ‘we’, associated with the beginnings of an appreciation of the collective phenomena of social norms and conventions. Conformity to majorities is increasingly revealed to be widespread in animal communities [120], but pre-school children begin to make explicit their recognition of these inherently collective phenomena; having observed demonstrations of some behavioural routine or rule, they will, for example, chide even a puppet seen to transgress it [121].

These and other experimental ‘laboratory’ studies are complemented by recent systematic and quantitative analyses of the nature and distribution of forms of social learning in children in the important hunter–gatherer populations referred to above (e.g. [120–126]). The latter study [126] documented that in BaYaka communities in the Congo, from infancy to early childhood, children learn mainly by directly observing and copying others' activities in categories such as foraging, tool use and cooking. Teaching by adults plays a more specific role in transmitting other less concrete forms of knowledge such as the social norms referred to above. From early childhood, social learning is reported to occur mainly in playgroups that are composed of mixed aged children, so overall, collective knowledge and cultural transmission can be conceived of as a cascade of information from adults to older children and thence to progressively younger ones. Lew-Levy *et al*. [125] confirmed this ‘cascade’ pattern in BaYaka children but not in Hadza children, so variations in cultural transmission networks merit further investigation among hunter–gatherer societies.

The latter study also reported that teaching was more prevalent between children than from adult to child, and the small multi-age playgroups of hunter–gatherers merit investigation as micro-society collectives in their own right. Experimental studies of small groups of children faced with opportunities for small-scale cumulative cultural changes have documented that they may function as collectives, displaying spontaneous teaching and cooperation [127] and achieving more cumulative progress than children acting on their own accord [128].

## 5. Social learning in swarm robotics

The term ‘social learning’ has been in use in robotics for about 30 years. Most often, it has referred to a very particular case of interaction between a robot and a human interlocutor, where the explicit programming of the robot's behaviours is replaced by learning through imitation or demonstration, or by exploring the environment while being partially guided by a human teacher [129,130]. However, when we instead consider transferring knowledge and/or skills between robots over the course of a lifetime, we turn to the field of multi-agent learning, and more specifically to what has been called ‘distributed online reinforcement learning’ for collective and swarm robotics [16]. This is a field of machine learning characterized by the presence of a large number of robots (dozens to thousands) whose computational and communication capacities are relatively limited in range and speed (peer-to-peer communication is slow with respect to the swarm size). This means that any information will diffuse only from one robot to its immediate neighbours, with possible delays owing to hardware and/or environmental contingencies. As a result, it is not possible to centralize the information required to perform learning and decision making for the whole population if the environment changes over time. It is also assumed that each robot embeds a decision making process, as well as an onboard evaluation function that estimates the quality of the produced behaviour with respect to a user-defined task. From the multi-agent learning perspective, this means that individual learning occurs in a competitive framework, where behavioural strategies (rather than the robots themselves) compete to invade the robot population.

Social learning in swarm robotics (SLSR) addresses a classical problem in this field, that of the difficulty of predicting the macroscopic behaviour observable *a posteriori* at the swarm scale on the basis of the microscopic behaviours produced by the robots (which are generally programmed by hand) [131]. Depending on the algorithm that is implemented, the social learning process will follow a dynamic that may be driven, for example, by the selective affinities between robots, or by the behaviour of the robots depending on the task at hand. The interest in SLSR started at the turn of the millennium, and was originally referred to as ‘embodied evolutionary robotics' [132,133]. Indeed, seminal works initially came as an extension of the methods used in evolutionary robotics [134–136], which proposes to use the so-called selection and variation operators used in evolutionary algorithms to automatically design the behavioural strategies of a single robot in order to address a user-defined task. More recently, the term ‘social learning’ has tended to spread, as it more accurately captures the nature of the problem addressed, which concerns the diffusion of skills or knowledge learned in a population of robots, rather than the nature of the algorithm used (i.e. evolutionary algorithms are but one possible option to implement social learning). Since the beginning, research on SLSR has been conducted both in simulation and, most notably, on real robots: although the learning dynamics can be quite complex, the fundamental algorithmic principles are relatively easy to implement on robots with limited hardware, which is typical of swarm robotics.

The classical process is as follows. The behaviour of each robot depends on a decision-making module, for example an artificial neural network, which defines the robot's behavioural strategy. The control parameters of this module determine how the robot acts (using its actuators) depending on the present situation (which is partially captured using on-board sensors). The problem is then to discover the relevant values for these parameters so that the robot performs as well as possible on a predefined task (for example, the foraging success during the last hour, which can be measured by the robot). In its simplest formulation, social learning is accomplished through two complementary actions carried out in parallel by each robot of the swarm: (i) by sharing with its neighbours part or all of its control parameters along with an estimation of the quality of its behaviour with respect to the task to be accomplished, and (ii) by updating its own control parameters with parameter values received from its neighbours that are best rated. Learning is thus accomplished in a distributed fashion by the diffusion of the ‘best’ control parameters through the population. Diffusion is subject to limited perturbations, which can modify (in a limited way) the transmitted information, and possibly allows exploration of new parameter values that may lead to ‘better’ (more adaptive) behaviours. We thus find two major Darwinian principles here: selection pressure favours the diffusion of the best values of control parameters, and random variations of limited amplitude allow the exploration of new behavioural strategies, which sometimes turn out to be better with respect to the user-defined task to be addressed.

The quantity and nature of the information exchanged between robots has an influence on the quality of their behaviour, as well as on the speed of diffusion of these behaviours in the swarm. In particular, the diffusion of a small amount of information allows exploitation of the possible recombinations between different behavioural strategies already present in the population [137], and presents an advantage with respect to the user-defined objective when compared to a simple imitation by copying [14,15]. Moreover, the way in which the information received by a robot is used has an influence not only on the improvement of performance [138,139], but also on the structure of the population itself, for example by allowing the specialization of subsets of the swarm to accomplish certain specific tasks [140,141]. Indeed, artificially limiting the pressure of task-oriented selection of the information shared by the encountered partners facilitates behavioural diversity among the robots, i.e. for any two given robots facing similar conditions, each may display a specific and possibly unique behaviour. Finally, an enforced preference towards original behaviours allows the encouragement of innovation and exploration, which in a complex environment is essential to discover efficient behaviours [142]. Even if the preference for original behaviours can be detrimental in the short term, it may favour the diffusion of innovative behaviour, which may emerge as a better choice with respect to the optimization problem initially posed.

Social learning algorithms, and more generally any learning algorithms distributed over a population of individuals whose learning both acts and depends on the social networks, are subject to two possibly antagonistic selective pressures. A given behaviour will diffuse in the population only (i) if it performs well with respect to the user-defined evaluation function, and (ii) if it is able to create opportunities for diffusion by meeting with other robots, which may also require learning basic survival skills not necessarily needed to fulfil the task given by the user [143–146]. The diffusion of behavioural strategies can be studied without any reference to the pursuit of a predefined goal; that is, in the absence of any user-defined objective function to be optimized. In that case, *natural* selection (as opposed to artificial goal-directed selection) drives learning towards strategies that are able to maintain the integrity of their robotic vehicle (e.g. energy autonomy, avoidance of breakdowns and accidents, etc.) while promoting opportunities to meet with other robots. As this process of natural selection occurs in a population of finite and fixed size, social learning algorithms follow a similar pattern to that of social learning in a population of living individuals, where behavioural strategies will change with each encounter solely based on interactions with one another and with the environment. In the absence of a measurable goal, SLSR can take into account, or even exploit, environmental contingencies to survive [147,148], sometimes and perhaps surprisingly taking advantage of the environment [149] or learning to forage in a cooperative manner, potentially increasing the chance of survival at both individual and population levels [150].

## 6. The scope of the current journal issue

We shall not offer a precis here for each contribution to this journal issue—readers can consult abstracts for that. Rather, we aim to indicate how the contributions relate to our overarching themes of collective knowledge, culture and cultural evolution, as indicated in the reviews above, as well as varied links between them.

**Figure 1.**
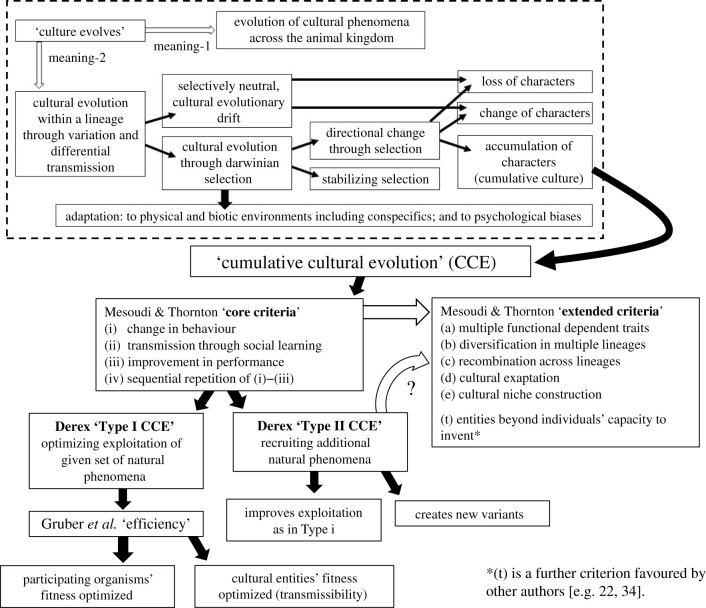
A taxonomy of distinctions between manifestations of ‘cultural evolution’. In the top, framed box (after [25]) are distinctions between two broad meanings of the assertion that ‘culture evolves’, followed by a dissection of forms of cultural evolution. The remainder of the figure illustrates distinctions within cumulative cultural evolution (CCE) due to [78,151,152], as discussed in the text. The white arrow between core and extended criteria for CCE indicates that the latter are potential variants within the category defined by the former. The white arrow between Type II CCE and the extended criteria indicates that whether the latter are causally dependent on the former is a remaining empirical question.

The first two contributions to the current issue have independently addressed the fundamental topic of how we should most productively conceptualize CCE. Both accept the ‘core criteria’ for CCE proposed by Mesoudi & Thornton [78] (figure 1) then dissect the resultant concept further, but in different ways. Derex [151] offers the broadest analysis, proposing a distinction between a Type I CCE and Type II CCE, on the basis of how these adaptively exploit the natural phenomena with which they are engaged. Type I optimizes exploitation of only a given set of phenomena, as in the case of the progressive efficiency of homing flights of pigeons cited above [32], and other cases of CCE in animals and humans (and presumably robot analogues too). Type II is distinguished by recruiting additional and different natural phenomena, as in such examples as the bow and arrow superseding thrown spears in human history.

Gruber *et al*. [152], focused on animal cases of CCE, argue that the core concept here is one of progressive enhancements in ‘efficiency’ of the system at stake. This appears to broadly map to Derex's Type I CCE, with both mapping to the ‘improvement in performance’ criterion of [78] (figure 1). However, readers must judge for themselves how close this mapping across [78,151,152] may be. The main thrust of Gruber *et al*. is to further distinguish efficiency in the currency of the performing organism's fitness, from efficiency in the currency of the cultural entity's fitness. The latter translates to the success of the entity (a cultural behaviour, or artefact) in transmission to others, an example of which would be a whale or bird song that outcompeted rivals in being faithfully adopted across populations and generations.

The subsequent five papers report empirical findings from studies of birds and mammals. Whiten *et al*. [153] describe several ways in which their recent research findings reveal links between collective knowledge and cultural transmission in chimpanzees. These include a sequence of events in one of three control groups of a transmission experiment, in which different individuals mastered different aspects of a novel form of tool use, then combined them to gain juice that was otherwise unobtainable. Other parts of the experiment demonstrated that this collective innovation, which built cumulatively on prior behaviour, then spread to others.

Wild *et al*. [154] likewise report experiments examining a cumulative progression that rested on combining behavioural elements, in this case in wild-living great tits. In a first phase, one of two foraging techniques introduced by trained model birds spread to become local traditions. A second pair of alternative techniques were later introduced in a similar way. The crucial question was then whether birds would learn to combine the two techniques when experimenters arranged that this gained better rewards, or was even essential to gain rewards, thus potentially exhibiting a form of CCE. The combination was indeed acquired and spread: however, detailed analysis suggested that birds learned the components socially, but combined them via individual learning, rather than socially learning the combined behaviour as a whole. This may appear to contrast with the chimpanzee experiment described above in which a complex form of tool use was achieved through combinations of inventions by different individuals, and once it appeared, was acquired by others as a whole. However, the experimental contexts were so different that this is best regarded as a possibility to explore through further systematic testing. In the meantime, the tit study yielded a host of instructive findings on linkages between collective actions and cultural transmission in avian communities.

Both the chimpanzee and tit progressions appear to fit Type I CCE [151] instantiated in foraging benefits to the practitioners [152]. One might question if the behavioural combination in the chimpanzee case also exploits additional physical phenomena (in this case, opening a valve), thus entering the domain of Type II CCE.

Two other studies, one avian, one mammalian, focus on the very different context of non-human vocal communication. Garland *et al*. [82] analyse striking cyclical changes in the songs of male humpback whales, evaluating these against the core criteria for CCE listed in figure 1. The ‘improvement’ implicating CCE in this case is instantiated by rises in objectively measured song complexity over several years, punctuated in turn by intermittent falls in complexity at times when radically new songs emerge. However, whether either of these changes benefits fitness (figure 1), for example by being more attractive to females, remains to be confirmed. Intriguing possibilities are that more complex songs become more transmissible, up to a point (figure 1, bottom), after which the revolutionary new songs become so, but for different reasons such as those connected with their relative simplicity, novelty or both.

In studies of CCE in the realm of bird song, Williams & Lachlan [155] suggest that both these aspects—benefits to practitioners, and benefits to the behaviour patterns in the form of transmissibility (figure 1)—may be in play in varying contexts. Detailed comparative analyses across different songbird studies reveal how evolutionary changes in song structures emerge through collective occurrences across populations in innovations, copying errors, cultural drift, learning biases and/or the selective advantages of some variants.

Beyond these case studies in animal communication, Kirby & Tamariz [156] demonstrate that cultural evolution can also explain the origins of one of the fundamental design features of human language, duality of patterning. Using simulations they show why some young emerging sign languages exhibit this feature to a greater or lesser extent, arguing that learning from other learners as opposed to adults radically increases the rate of evolution. They describe the system evolving to be *simpler*, particularly in environments with much horizontal transmission between children. It is important to note, however, that the simplicity that they are referring to here is the grammatical structure of the system as a whole (i.e. how much reuse there is of sub-elements in signals, and therefore how efficient the system is) which does not equate to simplicity in the signals themselves.

As we outlined earlier, work in robotics and machine intelligence has increasingly addressed both collective knowledge and social learning, yet there has been minimal cross-talk between this literature and that covering either animal of human research on these topics. In this issue, we have aimed to encourage such cross-dissemination via three articles [28–30] that review recent research embracing both learning and evolutionary processes that facilitate increasingly effective robotic action outcomes. Bredeche & Fontbonne [28] note that even in the case of social learning, evolving greater ‘efficiency’ (figure 1) of such outcomes is typically assessed only from the perspective of the individual. They show instead how social learning within swarms of robots can operate to enhance collective knowledge, with benefits at the group (swarm) level (which in turn may provide a net benefit to the individual robots that constitute it). CCE may arise from individuals' innovations, which can be recombined with existing behaviour, to improve performance at the level of the collective.

Conventions, inherently both collective and cultural phenomena, are a familiar example of such mutually beneficial behaviours in humans. Formaux *et al*. [157] review circumstantial evidence that conventions may also arise in a variety of non-human primates. The authors then go on to provide the first experimental evidence tracing the development of primate conventions that provide mutual benefits to the participants. The conventions that evolve in this research match three key, defining criteria of conventions: arbitrariness, efficiency and stability. Crucially, conventions stabilized even in the absence of visual access to a partner's behaviour, i.e. strictly as a consequence of shared reinforcement. This condition—where the solution could not have emerged without mutual input—highlights how the processes producing non-human conventions provide a particularly clear example of the role of the collective in generating, rather than simply providing a repository for, cultural innovation.

 Nonetheless, the solutions to coordination problems that emerge in [157] do not require advanced forms of social learning or cognition; in contrast, O'Madagain & Tomasello [158] review core forms of socio-cognitive sophistication that emerge in the course of human ontogeny and whose evolution facilitates a series of layers of mutually beneficial inter-individual coordination. These progressively extend to such phenomena as collaborative innovation and helping, and ‘epistemic pedagogy’, in which new technologies are transmitted as a result of being supported by a good explicit rationale, rather than owing to factors like frequency of use or past success. Whenever a new environmental challenge is experienced, it is argued that this human sharing of reasons for actions and beliefs can support powerful new adaptive responses, facilitating sophisticated cumulative culture.

Theory of mind, or ‘everyday mindreading' of others’ mental states is integral to these human achievements and through childhood develops from elementary to sophisticated. In their experiments with groups of robots, Winfield & Blackmore [29] ambitiously incorporate an elementary form of this kind of capacity, in the sense that the robots involved incorporate a ‘consequence engine’ that predicts (imagines) the outcomes of potential future actions with respect to self, but can also transmit it to other robots with the same internal machinery, which can thence represent (mindread) the first robot's predictions. These authors progress to experiments with these ‘story-telling’ robots via simpler experiments in which robots perceive and imitatively copy the two-dimensional movements of other robots in their environment. Through copying errors and other, contextual noise, this can lead to cultural evolution in the sense that different traditions of action configurations arise. Phenomena such as here labelled ‘theory of mind’ or ‘tradition’ may seem far from their complex counterparts in animals or humans, but there is arguably no better test of our understanding of these than to discover what must be implemented in an artificial agent in order to manifest even elementary forms.

Several articles in the issue further elucidate the forms of social and collective phenomena that shape cumulative culture. Schimmelpfennig *et al*. [159] review studies addressing how ‘cultural evolvability’ can be either enhanced or inhibited by variations in the three principal influences: social structure (e.g. connectedness), transmission fidelity and cultural trait diversity. They focus on, and model, the effects of core components in what they describe as the ‘paradox of diversity’ in collectives, wherein trait diversity can potentially constitute the most powerful enabler of evolvability, yet simultaneously creates barriers such as coordination challenges, so that the results in cumulative culture reflect a dynamic interplay across this array of variables.

Mommenejad [160] provides complementary perspectives on how different ‘social network topologies' shape collective memory and social transmission, drawing together insights from behavioural and cognitive experiments, neuroimaging of people's representations of social networks, and the emergence of collective phenomena in multi-agent machine-learning networks, the latter linking with the robotics articles in the issue.

Based on ethnographic, archaeological and genetic evidence, Migliano & Vinicius [93] propose that the evolutionary transition from an ape-like to a hunter–gatherer foraging niche engendered a unique social network structure facilitating CCE. They define this concept as a ‘social ratcheting’ that generates irreversible task specialization and division of labour uniquely found in humans, as the main adaptive consequence of human multilevel sociality. They argue that by splitting the burden of cultural knowledge across individuals in this way, social ratcheting has become a key component of human collective intelligence. Such conclusions from these real-world investigations converge encouragingly with reports from various laboratory and modelling studies in this issue [98,99,105,106,108,159,160].

There are further such links with research on culturally evolving learning among robots. Hart & Le Goff [30] report that evolving both the body and brain of robots can be enhanced by combining artificial evolution with either individual learning or a particular implementation of cultural learning. In that latter case, past experiences from previous robots are accumulated and stored in a repository. This accumulated culture can then be used to augment the evolutionary processes used to discover new robots, specifically by accelerating the learning mechanisms that are needed to fine-tune the controllers of new body plans. This may facilitate evolvability on more macroscopic scales, echoing such effects in the hunter–gatherer [93] and laboratory experimental contexts [99].

In turn, Romano *et al*. [161] use the advances in understanding derived from studies of contemporary humans outlined above as a lens with which to interpret the ancient past of our species. In this they focus on the past approximately 60 ka, a period over which the archaeological record has become rich, tracing the evolution of human technologies. What such analyses lack, to an extent inevitably, is comparably rich data on core phenomena reviewed in this theme issue, such as multi-scale social networks and forms of collective cognition. Romano *et al*. accordingly address our evolutionary cultural past through an integration of a diverse range of methodologies ranging from large scale inferences about population structures derived from the archaeological record to quantitative studies of hunter–gatherers (as in this issue: [93]), in concert with simulations and modelling.

## 7. Concluding remarks

In this issue we have assembled contributions that together provide rich analyses of collective knowledge and culture (particularly CCE), topics that have hitherto often been served by quite separate literatures. All articles address culture in some form, some focusing on cumulative culture in particular depth [151,152,156], others examining numerous different linkages between collective knowledge and culture [28–30,82,93,153–155,158–160]. We have sought to include articles that are strongly empirically anchored, and that span the diversity that exists in expression of these phenomena, from the simplest manifestations in animals and machines to the uniquely complex in humans.

It could be said that all culture embodies collective knowledge insofar as the mentality underlying some specific traditional behaviour is distributed across the population of interest. Recognizing this, but going far beyond it, articles in this issue address the significance of cases where collective knowledge exists only at the level of the collective, and not in any one individual. The partial or variant knowledge of different individuals that this implies may be distributed across a population in space, and/or over time, with subsequent combinations thence leading to innovations that can drive CCE [82,93,153–155,159,160]. Studies are progressively revealing that how these effects play out may be shaped by numerous interacting factors including the form of social structures and networks [93,159,160], relationships between individuals such as in degree of tolerance [153] or coordination [157], adaptive biases in model selection such as conformity [153,163], and the socio-cognitive capabilities of participant individuals, such as theory of mind [29,158] and inventiveness. The findings of the studies in this issue, alongside recent complementary explorations [91,162,164], provide a deepening understanding of the diverse manifestations of culture and its evolution in both natural and human-engineered contexts.

## Data accessibility

This article has no additional data.

## References

[1] Conradt L, Roper TJ. 2005 Consensus decision-making in animals. Trends Ecol. Evol. **20**, 449-456. (10.1016/j.tree.2005.05.008) 16701416

[2] Seeley TD. 2009 The wisdom of the hive. Cambridge, MA: Harvard University Press.

[3] Ward AJ, Sumpter DJ, Couzin ID, Hart PJ, Krause J. 2008 Quorum decision-making facilitates information transfer in fish shoals. Proc. Natl Acad. Sci. USA **105**, 6948-6953. (10.1073/pnas.0710344105) 18474860 PMC2383955

[4] Berdahl A, Torney CJ, Ioannou CC, Faria JJ, Couzin ID. 2013 Emergent sensing of complex environments by mobile animal groups. Science **339**, 574-576. (10.1126/science.1225883) 23372013

[5] Wechsler D. 1971 Concept of collective intelligence. Am. Psychol. **26**, 9-4-907.

[6] Surowicki J. 2004 The wisdom of crowds. London, UK: Random House.

[7] Muthukrishna M, Henrich J. 2016 Innovation in the collective brain. Phil. Trans. R. Soc. B **371**, 20150192. (10.1098/rstb.2015.0192) 26926282 PMC4780534

[8] Wilson R. 2005 Collective memory, group minds, and the extended mind thesis. Cogn. Process. **6**, 227-236. (10.1007/s10339-005-0012-z) 18239951

[9] Theiner G, Allen C, Goldstone RL. 2010 Recognizing group cognition. Cogn. Syst. Res. **11**, 378-395. (10.1016/j.cogsys.2010.07.002)

[10] Tomasello M. 2019 Becoming human: a theory of ontogeny. Cambridge, MA: Belknap Press.

[11] Kernbach S (ed.) 2013 Handbook of collective robotics: fundamentals and challenges, 1st edn. Singapore: Pan Stanford Publishing.

[12] Siciliano B, Khatib O (eds). 2016 Springer handbook of robotics. Berlin, Germany: Springer.

[13] Sahin E, Girgin S, Bayinder L, Turgut AE. 2008 Swarm robotics. In Swarm intelligence: introduction and applications (eds C Blum, D Merkle), pp. 87-100. Berlin, Germany: Spring-Verlag.

[14] Heinerman J, Drupsteen D, Eiben AE. 2015 Three-fold adaptivity in groups of robots: the effect of social learning. In Proc. Genetic and Evolutionary Computation Conf., July 2015, Madrid, Spain, pp. 177-183. New York, NY: ACM.

[15] Heinerman J, Rango M, Eiben AE. 2015 Evolution, individual learning and social learning in a swarm of real robots. In IEEE Symp. Seris Computational Intelligence (IEEE SSCI), December 2015, Cape Town, South Africa, pp. 1055-1062. New York, NY: IEEE.

[16] Bredeche N, Haasdijk E, Prieto A. 2018 Embodied evolution in collective robotics: a review. Front. Robot. AI **5**, 12. (10.3389/frobt.2018.00012) 33500899 PMC7806005

[17] Hamann H. 2018 Swarm robotics: a formal approach. Berlin, Germany: Springer.

[18] Mulgan G. 2018 Big mind: how collective intelligence can change our world. Princeton, NJ: Princeton University Press.

[19] Tylor EB. 1871 Primitive culture: researches into the development of mythology, philosophy, religion, art and custom. London, UK: John Murray.

[20] Medawar P. 1977 Unnatural science. In The strange case of the spotted mouse and other classic essays on science, pp. 144-161. Oxford, UK: Oxford University Press.

[21] Ridley M. 2015 The evolution of everything: how new ideas emerge. London, UK: Fourth Estate.

[22] Henrich J. 2016 The secret of our success: how culture is driving evolution, domesticating our species, and making us smarter. Princeton, NJ: Princeton University Press.

[23] Whitehead H, Rendell L. 2015 The cultural lives of whales and dolphins. Chicago, IL: Chicago University Press.

[24] Aplin LM. 2019 Culture and cultural evolution in birds: a review of the evidence. Anim. Behav. **147**, 179-187. (10.1016/j.anbehav.2018.05.001)

[25] Whiten A. 2019 Cultural evolution in animals. Ann. Rev. Ecol. Evol. Syst. **50**, 27-48. (10.1146/annurev-ecolsys-110218-025040)

[26] Whiten A. 2021 The burgeoning reach of animal culture. Science **372**, eabe5414. (10.1126/science.abe6514) 33795431

[27] Lachlan RF, Whiten A. 2020 Cultural evolution in non-human animals. In Oxford bibliographies in evolutionary biology (ed. D Futuyma). Oxford, UK: Oxford University Press. (10.1093/OBO/9780199941728-0129)

[28] Bredeche N, Fontbonne N. 2021 Social learning in swarm robotics. Phil. Trans. R. Soc. B **377**, 20200309. (10.1098/rstb.2020.0309) 34894730 PMC8666954

[29] Winfield AFT, Blackmore S. 2021 Experiments in artificial culture: from noisy imitation to storytelling robots. Phil. Trans. R. Soc. B **377**, 20200323. (10.1098/rstb.2020.0323) 34894733 PMC8666905

[30] Hart E, Le Goff LK. 2021 Artificial evolution of robot bodies and control: on the interaction between evolution, learning and culture. Phil. Trans. R. Soc. B **377**, 20210117. (10.1098/rstb.2021.0117) 34894727 PMC8666908

[31] Biro D, Sasaki T, Portugal SJ. 2016 Bringing a time-depth perspective to collective animal behaviour. Trends Ecol. Evol. **31**, 550-562. (10.1016/j.tree.2016.03.018) 27105543

[32] Sasaki T, Biro D. 2017 Cumulative culture can emerge from collective intelligence in animal groups. Nat. Commun. **8**, 15049. (10.1038/ncomms15049) 28416804 PMC5399285

[33] Jesmer BR et al. 2018 Is ungulate migration culturally transmitted? Evidence of social learning from translocated animals. Science **361**, 1023-1025. (10.1126/science.aat0985) 30190405

[34] Tennie C, Call J, Tomasello M. 2009 Ratcheting up the ratchet: on the evolution of cumulative culture. Phil. Trans. R. Soc. B **364**, 2405-2415. (10.1098/rstb.2009.0052) 19620111 PMC2865079

[35] Vicsek T, Zafeiris A. 2012 Collective motion. Phys. Rep. **517**, 71-140. (10.1016/j.physrep.2012.03.004)

[36] Ballerini M et al. 2008 Interaction ruling animal collective behavior depends on topological rather than metric distance: evidence from a field study. Proc. Natl Acad. Sci. USA **105**, 1232-1237. (10.1073/pnas.0711437105) 18227508 PMC2234121

[37] Katz Y, Tunstrøm K, Ioannou CC, Huepe C, Couzin I. 2011 Inferring the structure and dynamics of interactions in schooling fish. Proc. Natl Acad. Sci. USA **108**, 18 720-18 725. (10.1073/pnas.1107583108) 21795604 PMC3219116

[38] Latty T, Ramsch K, Ito K, Nakagaki T, Sumpter DJT, Middendorf M, Beekman M. 2011 Structure and formation of ant transportation networks. J. R. Soc. Interface **8**, 1298-1306. (10.1098/rsif.2010.0612) 21288958 PMC3140716

[39] Khuong A, Gautrais J, Perna A, Sbaï C, Combe M, Kuntz P, Jost C, Theraulaz G. 2016 Stigmergic construction and topochemical information shape ant nest architecture. Proc. Natl Acad. Sci. USA **113**, 1303-1308. (10.1073/pnas.1509829113) 26787857 PMC4747701

[40] Reid C, Lutz MJ, Powell S, Kao AB, Couzin ID, Garnier S. 2015 Army ants dynamically adjust living bridges in response to a cost–benefit trade-off. Proc. Natl Acad. Sci. USA **112**, 15 113-15 118. (10.1073/pnas.1512241112) PMC4679032 26598673

[41] Buck J. 1988 Synchronous rhythmic flashing of fireflies. II. Q. Rev. Biol. **63**, 265-289. (10.1086/415929) 3059390

[42] Herbert-Read JE, Buhl J, Hu F, Ward AJW, Sumpter DJT. 2015 Initiation and spread of escape waves within animal groups. R. Soc. Open Sci. **2**, 140355. (10.1098/rsos.140355) 26064630 PMC4448869

[43] Conradt L, Roper TJ. 2003 Group decision-making in animals. Nature **421**, 155-158. (10.1038/nature01294) 12520299

[44] Camazine S, Deneubourg J-L, Franks NR, Sneyd J, Theraulaz G, Bonabeau E. 2001 Self-organisation in biological systems. Princeton, NJ: Princeton University Press.

[45] Couzin ID. 2009 Collective cognition in animal groups. Trends Cogn. Sci. **13**, 36-43. (10.1016/j.tics.2008.10.002) 19058992

[46] Couzin ID. 2007 Collective minds. Nature **445**, 715. (10.1038/445715a) 17301775

[47] Couzin ID, Krause J, Franks NR, Levin SA. 2005 Effective leadership and decision-making in animal groups on the move. Nature **433**, 513-516. (10.1038/nature03236) 15690039

[48] Strandburg-Peshkin A, Farine DR, Couzin ID, Crofoot MC. 2015 Shared decision-making drives collective movement in wild baboons. Science **348**, 1358-1361. (10.1126/science.aaa5099) 26089514 PMC4801504

[49] Biro D, Sumpter DJ, Meade J, Guilford T. 2006 From compromise to leadership in pigeon homing. Curr. Biol. **16**, 2123-2128. (10.1016/j.cub.2006.08.087) 17084696

[50] Papageorgiou D. 2021 Collective movement and social decision-making in the vulturine guineafowl (*Acryllium vulturinum*). PhD Thesis, University of Konstanz, Konstanz, Germany.

[51] Seeley T, Visscher PK. 2004 Quorum sensing during nest site selection by honey-bee swarms. Behav. Ecol. **56**, 594-601. (10.1007/s00265-004-0814-5)

[52] Pratt SC, Mallon EB, Sumpter DJT, Franks NR. 2002 Quorum sensing, recruitment, and collective decision-making during colony emigration by the ant *Leptothorax albipennis*. Behav. Ecol. Sociobiol. **52**, 117-127. (10.1007/s00265-002-0487-x)

[53] Dyer JRG, Ioannou CC, Morrell LJ, Croft DP, Couzin ID, Waters DA, Krause J. 2008 Consensus decision making in human crowds. Anim. Behav. **75**, 461-470. (10.1016/j.anbehav.2007.05.010)

[54] Conradt L, Krause J, Couzin ID, Roper TJ. 2009 ‘Leading according to need’ in self-organizing groups. Am. Nat. **173**, 304-312. (10.1086/596532) 19199520

[55] Fischhoff IR, Sundaresan SR, Cordingley J, Larkin HM, Sellier MJ, Rubenstein DI. 2007 Social relationships and reproductive state influence leadership roles in movements of plains zebra, *Equus burchellii*. Anim. Behav. **73**, 825-831. (10.1016/j.anbehav.2006.10.012)

[56] Webster MM, Whalen A, Laland KN. 2017 Fish pool their experience to solve problems collectively. Nat. Ecol. Evol. **1**, 0135. (10.1038/s41559-017-0135) 28812697

[57] Laland KN, Atton N, Webster MM. 2011 From fish to fashion: experimental and theoretical insights into the evolution of culture. Phil. Trans. R. Soc. B **366**, 958-968. (10.1098/rstb.2010.0328) 21357218 PMC3049094

[58] Hoppitt W, Laland KN. 2013 Social learning: an introduction to mechanism, models and models. Princeton, NJ: Princeton University Press.

[59] Worden BD, Papaj DR. 2005 Flower choice copying in bumblebees. Biol. Lett. **1**, 504-507. (10.1098/rsbl.2005.0368) 17148244 PMC1626359

[60] Alem S, Perry CJ, Zhu X, Loukola OJ, Ingraham T, Søvik E, Chittka L. 2016 Associative mechanisms allow for social learning and cultural transmission of string pulling in an insect. PLoS Biol. **14**, e1002564. (10.1371/journal.pbio.1002564) 27701411 PMC5049772

[61] Danchin É et al. 2018 Cultural flies: conformist social learning in fruit flies predicts long-lasting mate-choice traditions. Science **362**, 1025-1030. (10.1126/science.aat1590) 30498121

[62] Aplin LM, Farine DR, Morand-Ferron J, Cockburn A, Thornton A, Sheldon BC. 2015 Experimentally induced innovations lead to persistent culture via conformity in wild birds. Nature **518**, 538-541. (10.1038/nature13998) 25470065 PMC4344839

[63] Allen JA, Weinrich M, Hoppitt W, Rendell L. 2013 Network-based diffusion analysis reveals cultural transmission of lobtail feeding in humpback whales. Science **340**, 485-488. (10.1126/science.1231976) 23620054

[64] Whiten A, Goodall J, McGrew WC, Nishida T, Reynolds V, Sugiyama Y, Tutin CEG, Wrangham RW, Boesch C. 1999 Cultures in chimpanzees. Nature **399**, 682-685. (10.1038/21415) 10385119

[65] Boesch C et al. 2020 Chimpanzee ethnography reveals unexpected cultural diversity. Nat. Hum. Behav. **4**, 910-916. (10.1038/s41562-020-0890-1) 32451479

[66] Marler P, Tamura M. 1964 Song ‘dialects’ in three populations of white-crowned sparrows. Science **146**, 1483-1486. (10.1126/science.146.3650.1483) 14208581

[67] Catchpole CK, Slater PJB. 2008 Bird song: biological themes and variations, 2nd edn. Cambridge, UK: Cambridge University Press.

[68] Perry S et al. 2003 Social conventions in white-face capuchins monkeys: evidence for behavioral traditions in a neotropical primate. Curr. Anthropol. **44**, 241-268. (10.1086/345825)

[69] van Leeuwen EJC, Cronin KA, Haun SBM. 2014 A group specific arbitrary tradition in chimpanzees. Anim. Cogn. **17**, 1421-1425. (10.1007/s10071-014-0766-8) 24916739

[70] Samuni L, Wegdell F, Surbeck M. 2020 Behavioral diversity of bonobo prey preferences as a potential cultural trait. Elife **9**, e59191. (10.7554/eLife.59191) 32869740 PMC7462605

[71] van de Waal E, Borgeaud C, Whiten A. 2013 Potent social learning and conformity shape a wild primate's foraging decisions. Science **340**, 483-485. (10.1126/science.1232769) 23620053

[72] Mueller T, O'Hara RB, Converse SJ, Urbanek RP, Fagan WF. 2013 Social learning of migratory performance. Science **341**, 999-1002. (10.1126/science.1237139) 23990559

[73] Carroll EL, Baker CS, Watson M, Alderman R, Bannister J, Gaggiotti OE, Gröcke DR, Patenaude N, Harcourt R. 2015 Cultural traditions across a migratory network shape the genetic structure of southern right whales around Australia and New Zealand. Sci. Rep. **5**, 16182. (10.1038/srep16182) 26548756 PMC4637828

[74] Whiten A, van de Waal E. 2018 The pervasive role of social learning in primate lifetime development. Behav. Ecol. Sociobiol. **72**, 80. (10.1007/s00265-018-2489-3) 29755181 PMC5934467

[75] Schuppli C, van Schaik CP. 2019 Animal cultures: how we've only seen the tip of the iceberg. Evol. Hum. Sci. **1**, e2. (10.1017/ehs.2019.1) PMC10427297 37588402

[76] Hobaiter C, Poiset T, Zuberbuhler K, Hoppitt W, Gruber T. 2014 Social network analysis shows direct evidence for social transmission of tool use in wild chimpanzees. PLoS Biol. **12**, e1001960. (10.1371/journal.pbio.1001960) 25268798 PMC4181963

[77] Falótico T, Proffitt T, Ottoni EB, Staff RA, Haslam M. 2019 Three thousand years of wild capuchin tool use. Nat. Ecol. Evol. **3**, 1034-1038. (10.1038/s41559-019-0904-4) 31235926

[78] Mesoudi A, Thornton A. 2018 What is cumulative cultural evolution? Proc. R. Soc. B **285**, 20180712. (10.1098/rspb.2018.0712) PMC6015846 29899071

[79] Claidiere N, Smith K, Kirby S, Fagot J. 2014 Cultural evolution of systematically structured behaviour in a non-human primate. Proc. R. Soc. B **281**, 20141541. (10.1098/rspb.2014.1541) PMC4240982 25377450

[80] Kirby S, Tamariz M, Cornish H, Smith K. 2015 Compression and communication in the cultural evolution of linguistic structure. Cognition **141**, 87-102. (10.1016/j.cognition.2015.03.016) 25966840

[81] Allen JA, Garland EC, Dunlop RA, Noad MJ. 2018 Cultural revolutions reduce complexity in the songs of humpback whales. Proc. R. Soc. B **285**, 20182088. (10.1098/rspb.2018.2088) PMC6253384 30464066

[82] Garland EC, Garrigue C, Noad MJ. 2021 When does cultural evolution become cumulative culture? A case study of humpback whale song. Phil. Trans. R. Soc. B **377**, 20200313. (10.1098/rstb.2020.0313) 34894734 PMC8666910

[83] Galton F. 1907 Vox populi. Nature **1949**, 450-451. (10.1038/075450a0)

[84] Malone TW, Bernstein MS (eds) 2015 Handbook of collective intelligence. Cambridge, MA: MIT Press.

[85] Richerson PJ, Christiansen MH (eds) 2013 Cultural evolution: society, technology, language and religion. Cambridge, MA: MIT Press.

[86] Schleicher A. 1869 Darwin tested by the science of language. London, UK: JC Hoten.

[87] Darwin C. 1871 The descent of Man and selection in relation to sex. London, UK: Murray.

[88] Pitt-Rivers AHL-F. 1906 The evolution of culture. Oxford, UK: Clarendon Press.

[89] Cavalli-Sforza LL, Feldman MW. 1981 Cultural transmission and evolution: a quantitative approach. Princeton, NJ: Princeton University Press. 7300842

[90] Boyd R, Richerson P. 1985 Culture and the evolutionary process. Chicago, IL: University of Chicago Press.

[91] Derex M, Mesoudi A. 2020 Cumulative cultural evolution within evolving population structures. Trends Cogn. Sci. **24**, 654-667. (10.1016/j.tics.2020.04.005) 32466991

[92] Boyd R, Silk J. 2020 How humans evolved, 9th edn. New York, NY: Norton and Co.

[93] Migliano AB, Vinicius L. 2021 The origins of human cumulative culture: from the foraging niche to collective intelligence. Phil. Trans. R. Soc. B **377**, 20200317. (10.1098/rstb.2020.0317) 34894737 PMC8666907

[94] Hill KR, Wood BM, Baggio J, Hurtado AM, Boyd RT. 2014 Hunter-gatherer inter-band interaction rates: implications for cumulative culture. PLoS ONE **9**, e102806. (10.1371/journal.pone.0102806) 25047714 PMC4105570

[95] Powell A, Shennan S, Thomas MG. 2009 Late Pleistocene demography and the appearance of modern human behavior. Science **324**, 1298-1301. (10.1126/science.1170165) 19498164

[96] Migliano AB et al. 2017 Characterization of hunter-gatherer networks and implications for cumulative culture. Nat. Hum. Behav. **1**, 0043. (10.1038/s41562-016-0043)

[97] Salali GD et al. 2016 Knowledge-sharing networks in hunter-gatherers and the evolution of cumulative culture. Curr. Biol. **26**, 2516-2521. (10.1016/j.cub.2016.07.015) 27618264

[98] Derex M, Perrault C, Boyd R. 2016 Partial connectivity increases cultural accumulation within groups. Proc. Natl Acad. Sci. USA **113**, 2982-2987. (10.1073/pnas.1518798113) 26929364 PMC4801235

[99] Derex M, Perreault C, Boyd R. 2018 Divide and conquer: intermediate levels of population fragmentation maximize cultural accumulation. Phil. Trans. R. Soc. B **373**, 20170062. (10.1098/rstb.2017.0062) 29440527 PMC5812974

[100] Migliano AB et al. 2020 Hunter-gatherer multilevel sociality accelerates cumulative cultural evolution. Sci. Adv. **6**, eaax5913. (10.1126/sciadv.aax5913) 32158935 PMC7048420

[101] Sikora M et al. 2017 Ancient genomes show social and reproductive behavior of early 708 Upper Paleolithic foragers. Science **358**, 659-662. (10.1126/science.aao1807) 28982795

[102] Brooks AS et al. 2018 Long-distance stone transport and pigment use in the earliest Middle Stone Age. Science **360**, 90-94. (10.1126/science.aao2646) 29545508

[103] Romano V, Lozano S, Fernández-López de Pablo J. 2020 A multi-level analytical framework for the study of cultural evolution in prehistoric hunter-gatherer societies. Biol. Rev. **95**, 1020-1035. (10.1111/brv.12599) 32237025 PMC7383820

[104] Caldwell CA, Millen AE. 2008 Studying cumulative culture in the laboratory. Phil. Trans. R. Soc. B **363**, 3529-3539. (10.1098/rstb.2008.0133) 18799419 PMC2607341

[105] Muthukrishna M, Shulman M, Vasilescu V. 2013 Sociality influences cultural complexity. Proc R. Soc. B **281**, 20132511. (10.1098/rspb.2013.2511) PMC3843838 24225461

[106] Kempe M, Mesoudi A. 2014 An experimental demonstration of the effect of group size on cultural accumulation. Evol. Hum. Behav. **35**, 285-290. (10.1016/j.evolhumbehav.2014.02.009)

[107] Henrich J, Broesch J. 2011 On the nature of cultural transmission networks: evidence from Fijian villages for adaptive learning biases. Phil. Trans. R. Soc. B **366**, 1139-1148. (10.1098/rstb.2010.0323) 21357236 PMC3049092

[108] Derex M, Boyd R. 2015 The foundations of the cultural niche. Nat. Commun. **6**, 8398. (10.1038/ncomms9398) 26400015 PMC4598620

[109] Wisdom TN, Song X, Goldstone RL. 2013 Social learning strategies in networked groups. Cogn. Sci. **37**, 1383-1425. (10.1111/cogs.12052) 23845020

[110] Goldstone RL, Wisdom TN, Roberts ME, Frey S. 2013 Learning along with others. In Psychology of learning and motivation, vol. 58 (ed. BH Ross), pp. 1-45. San Diego, CA: Elsevier.

[111] Tamariz M. 2017 Experimental studies of the cultural evolution of language. Annu. Rev. Linguist. **3**, 389-407. (10.1146/annurev-linguistics-011516-033807)

[112] Smith ADM. 2014 Models of language evolution and change. Wiley Interdiscip. Rev.: Cogn. Sci. **5**, 281-293. (10.1002/wcs.1285) 26308563

[113] Culbertson J, Schuler K. 2019 Artificial language learning in children. Annu. Rev. Linguist. **5**, 353-373. (10.1146/annurev-linguistics-011718-012329)

[114] Kirby S, Griffiths T, Smith K. 2014 Iterated learning and the evolution of language. Curr. Opin. Neurobiol. **28**, 108-114. (10.1016/j.conb.2014.07.014) 25062470

[115] Kemp C, Regier T. 2012 Kinship categories across languages reflect general communicative principles. Science **336**, 1049-1054. (10.1126/science.1218811) 22628658

[116] Newmeyer FJ. 2002 Uniformitarian assumptions and language evolution research. In The transition to language (ed. Alison Wray), pp. 359-375. Oxford, UK: Oxford University Press.

[117] Lupyan G, Dale R. 2010 Language structure is partly determined by social structure. PLoS ONE **5**, e8559. (10.1371/journal.pone.0008559) 20098492 PMC2798932

[118] Raviv L, Meyer A, Lev-Ari S. 2019 Larger communities create more systematic languages. Proc. R. Soc. B **286**, 20191262. (10.1098/rspb.2019.1262) PMC6661353 31311478

[119] Atkinson M, Smith K, Kirby S. 2018 Adult learning and language simplification. Cogn. Sci. **42**, 2818-2854. (10.1111/cogs.12686) 30320460 PMC6492256

[120] Whiten A. 2019 Conformity and over-imitation: an integrative review of variant forms of hyper-reliance on social learning. Adv. Study Behav. **51**, 31-75. (10.1016/bs.asb.2018.12.003)

[121] Rakoczy H, Warneken F, Tomasello M. 2008 The sources of normativity: young children's awareness of the normative structure of games. Dev. Psychol. **44**, 875. (10.1037/0012-1649.44.3.875) 18473651

[122] Hewlett BS, Fouts HN, Boyette AH, Hewlett BL. 2011 Social learning among Congo Basin hunter-gatherers. Phil. Trans. R. Soc. B **366**, 1168-1178. (10.1098/rstb.2010.0373) 21357239 PMC3049106

[123] Lew-Levy S, Lavi N, Reckin R, Cristóbal-Azkarate J, Ellis-Davies K. 2017 How do hunter-gatherer children learn social and gender norms? A meta-ethnographic review. Cross-Cult. Res. **52**, 213-255. (10.1177/1069397117723552) PMC5662667 28994008

[124] Lew-Levy S, Reckin R, Lavi N, Cristóbal-Azkarate J, Ellis-Davies K. 2017 How do hunter-gatherer children learn subsistence skills?: a meta-ethnographic review. Hum. Nat. **28**, 367-394. (10.1007/s12110-017-9302-2) 28994008 PMC5662667

[125] Lew-Levy S, Kissler SM, Boyette AH, Crittenden AN, Mabulla IA, Hewlett BS. 2020 Who teaches children to forage? Exploring the primacy of child-to-child teaching among Hadza and BaYaka hunter-gatherers of Tanzania and Congo. Evol. Hum. Behav. **41**, 12-22. (10.1016/j.evolhumbehav.2019.07.003)

[126] Salali GD, Chaudhary N, Bouer J, Thompson J, Vinicius L, Migliano AB. 2019 Development of social learning and play in BaYaka hunter-gatherers of Congo. Sci. Rep. **9**, 11080. (10.1038/s41598-019-47515-8) 31367002 PMC6668464

[127] Dean LG, Kendal RL, Schapiro SJ, Thierry B, Laland KN. 2012 Identification of the social and cognitive processes underlying human cumulative culture. Science **335**, 1114-1118. (10.1126/science.1213969) 22383851 PMC4676561

[128] McGuigan N, Burdett E, Burgess V, Dean L, Lucas A, Vale G, Whiten A. 2017 Innovation and social transmission in experimental micro-societies: exploring the scope of cumulative culture in young children. Phil. Trans. R. Soc. B **372**, 20160425. (10.1098/rstb.2016.0425) 29061897 PMC5665812

[129] Nehaniv CL, Dautenhahn K (eds) 2007 Imitation and social learning in robots, humans and animals: behavioural, social and communicative dimensions. Cambridge, UK: Cambridge University Press.

[130] Breazeal C, Buchsbaum D, Gray J, Gatenby D, Blumberg B. 2005 Learning from and about others: towards using imitation to bootstrap the social understanding of others by robots. Artif. Life **11**, 1-32. (10.1162/1064546053278955) 15811219

[131] Hamann H. 2018 Swarm robotics: a formal approach. Berlin, Germany: Springer.

[132] Ficici SG, Watson RA, Pollack JB. 1999 Embodied evolution: a response to challenges in evolutionary robotics. In Proc. of the Eighth European workshop on Learning Robots, September 1999, Lausanne, Switzerland. Berlin, Germany: Springer-Verlag.

[133] Watson RA, Ficici SG, Pollack JB. 2002 Embodied evolution: distributing an evolutionary algorithm in a population of robots. Robot. Auton. Syst. **39**, 1-18. (10.1016/S0921-8890(02)00170-7)

[134] Harvey I, Husbands P, Cliff D, Thompson A, Jakobi N. 1997 Evolutionary robotics: the Sussex approach. Robot. Auton. Syst. **20**, 205-224. (10.1016/S0921-8890(96)00067-X)

[135] Nolfi S, Floreano D. 2000 Evolutionary robotics: the biology, intelligence, and technology of self-organizing machines. Cambridge, MA: MIT Press.

[136] Doncieux S, Bredeche N, Mouret J-B, Eiben AE. 2015 Evolutionary robotics: what, why, and where to. Front. Robot. AI **2**, 4. (10.3389/frobt.2015.00004)

[137] Fontbonne N, Dauchot O, Bredeche N. 2020 Distributed on-line learning in swarm robotics with limited communication bandwidth. In Proc. of the IEEE Congress on Evolutionary Computation (CEC), July 2020, Glasgow, UK, pp. 1-8. New York, NY: IEEE.

[138] Fernández Pérez I, Boumaza A, Charpillet F. 2014 Comparison of selection methods in on-line distributed evolutionary robotics. In ALIFE 14: The Fourteenth Int. Conf. on the Synthesis and Simulation of Living Systems, July 2014, New York, NY, pp. 282-289. Cambridge, MA: MIT Press.

[139] Hart E, Steyven A, Paechter B. 2015 Improving survivability in environment-driven distributed evolutionary algorithms through explicit relative fitness and fitness proportionate communication. In Proc. of the Genetic and Evolutionary Computation Conf., July 2015, Madrid, Spain, pp. 169-176. New York, NY: ACM.

[140] Trueba P, Prieto A, Bellas F, Caamaño P, Duro RJ. 2013 Specialization analysis of embodied evolution for robotic collective tasks. Robot. Auton. Syst. **61**, 682-693. (10.1016/j.robot.2012.08.005)

[141] Montanier J-M, Carrignon S, Bredeche N. 2016 Behavioural specialisation in embodied evolutionary robotics: why so difficult? Front. Robot. AI **3**, number 38.

[142] Hart E, Steyven A, Paechter B. 2018 Evolution of a functionally diverse swarm via a novel decentralised quality-diversity algorithm. In Proc. of the Genetic and Evolutionary Computation Conf., July 2018, Kyoto, Japan, pp. 101-108. New York, NY: ACM.

[143] Haasdijk E, Bredeche N, Eiben AE. 2014 Combining environment-driven adaptation and task-driven optimisation in evolutionary robotics. PLoS ONE **9**, e98466. (10.1371/journal.pone.0098466) 24901702 PMC4047010

[144] Haasdijk E. 2015 Combining conflicting environmental and task requirements in evolutionary robotics. In Proc. of the IEEE Int. Conf. on Self-Adaptive and Self-Organizing Systems, September 2015, Cambridge, MA, pp. 131-137. New York, NY: IEEE.

[145] Haasdijk E, Eigenhuis F. 2016 Increasing reward in biased natural selection decreases task performance. In Proc. of the 15th Int. Conf. on Artificial Life, July 2020, Denver, CO, pp. 314-322. Cambridge, MA: MIT Press.

[146] Steyven A, Hart E, Paechter B. 2016 Understanding environmental influence in an open-ended evolutionary algorithm. In Int. Conf. on Parallel Problem Solving from Nature. Lecture Notes in Computer Science, vol. 9921, pp. 921-931: Berlin, Germany: Springer.

[147] Bredeche N, Montanier J-M. 2010 Environment-driven Embodied Evolution in a Population of Autonomous Agents. In 11th Int. Conf. on Parallel Problem Solving From Nature. PPSN XI, Part II, LNCS 6239, pp. 290-299. Berlin, Germany: Springer.

[148] Bredeche N, Montanier JM, Liu W, Winfield AF. 2012 Environment-driven distributed evolutionary adaptation in a population of autonomous robotic agents. Math. Comput. Model. Dyn. Syst. (MCMDS) **18**, 01-129. (10.1080/13873954.2011.601426)

[149] Bredeche N. 2014 Embodied evolutionary robotics with large numbers of robots. In Proc. of the Int. Conf. on the Synthesis and Simulation of Living Systems (ALIFE 14), July 2014, New York, NY, pp. 272-273. Cambridge, MA: MIT Press.

[150] Montanier J-M, Bredeche N. 2011 Surviving the tragedy of commons: emergence of altruism in a population of evolving autonomous agents. In Proc. of the European Conf. on Artificial Life, August 2011, Paris, France, pp. 550-557. Cambridge, MA: MIT Press.

[151] Derex M. 2021 Human cumulative culture and the exploitation of natural phenomena. Phil. Trans. R. Soc. B **377**, 20200311. (10.1098/rstb.2020.0311) 34894732 PMC8666902

[152] Gruber T, Chimento M, Aplin LM, Biro D. 2021 Efficiency fosters cumulative culture across species. Phil. Trans. R. Soc. B **377**, 20200308. (10.1098/rstb.2020.0308) 34894729 PMC8666915

[153] Whiten A, Harrison RA, McGuigan N, Vale GL, Watson SK. 2021 Collective knowledge and the dynamics of culture in chimpanzees. Phil. Trans. R. Soc. B **377**, 20200321. (10.1098/rstb.2020.0321) 34894742 PMC8666901

[154] Wild S, Chimento M, McMahon K, Farine DR, Sheldon BC, Aplin LM. 2021 Complex foraging behaviours in wild birds emerge from social learning and recombination of components. Phil. Trans. R. Soc. B **377**, 20200307. (10.1098/rstb.2020.0307) 34894740 PMC8666913

[155] Williams H, Lachlan RF. 2021 Evidence for cumulative cultural evolution in bird song. Phil. Trans. R. Soc. B **377**, 20200322. (10.1098/rstb.2020.0322) 34894731 PMC8666912

[156] Kirby S, Tamariz M. 2021 Cumulative cultural evolution, population structure and the origin of combinatoriality in human language. Phil. Trans. R. Soc. B **377**, 20200319. (10.1098/rstb.2020.0319) 34894728 PMC8666903

[157] Formaux A, Paleressompoulle D, Fagot J, Claidière N. 2021 The experimental emergence of convention in a non-human primate. Phil. Trans. R. Soc. B **377**, 20200310. (10.1098/rstb.2020.0310) 34894743 PMC8666916

[158] O'Madagain C, Tomasello M. 2021 Shared intentionality, reason-giving and the evolution of human culture. Phil. Trans. R. Soc. B **377**, 20200320. (10.1098/rstb.2020.0320) 34894741 PMC8666906

[159] Schimmelpfennig R, Razek L, Schnell E, Muthukrishna M. 2021 Paradox of diversity in the collective brain. Phil. Trans. R. Soc. B **377**, 20200316. (10.1098/rstb.2020.0316) 34894736 PMC8666911

[160] Momennejad I. 2021 Collective minds: social network topology shapes collective cognition. Phil. Trans. R. Soc. B **377**, 20200315. (10.1098/rstb.2020.0315) 34894735 PMC8666914

[161] Romano V, Lozano S, Fernández-López de Pablo J. 2021 Reconstructing social networks of Late Glacial and Holocene hunter–gatherers to understand cultural evolution. Phil. Trans. R. Soc. B **377**, 20200318. (10.1098/rstb.2020.0318) 34894739 PMC8666909

[162] Creanza N, Kolodny O, Feldman MW. 2017 Greater than the sum of its parts? Modelling population contact and interaction of cultural repertoires. J. R. Soc. Interface **14**, 20170171. (10.1098/rsif.2017.0171) 28468920 PMC5454306

[163] Kendal R et al. 2018 Social learning strategies: bridge-building between disciplines. Trends Ecol. Evol. **22**, 651-655. 10.1016/j.tics.2018.04.003 29759889

[164] Singh M et al. 2017 Beyond social learning. Phil. Trans. R. Soc. B **376**, 20200050. (10.1098/rstb.2020.0050) PMC8126463 33993759

