# Correction to ‘The emergence of collective knowledge and cumulative culture in animals, humans and machines’

**DOI:** 10.1098/rstb.2022.0020

**Published:** 2022-05-09

**Authors:** Andrew Whiten, Dora Biro, Nicolas Bredeche, Ellen C. Garland, Simon Kirby

*Phil. Trans. R. Soc. B* **377**, 20200306. Published 13 December 2021. (doi:10.1098/rstb.2020.0306)

The originally published version of this paper showed an error in figure 1. In the box titled ‘Mesoudi and Thornton “extended criteria”’, the six criteria were incorrectly grouped together as *a*–*f*, when the final point should have been separated. The corrected figure is shown below. This has also been corrected on the publisher's website.

**Figure 1.**
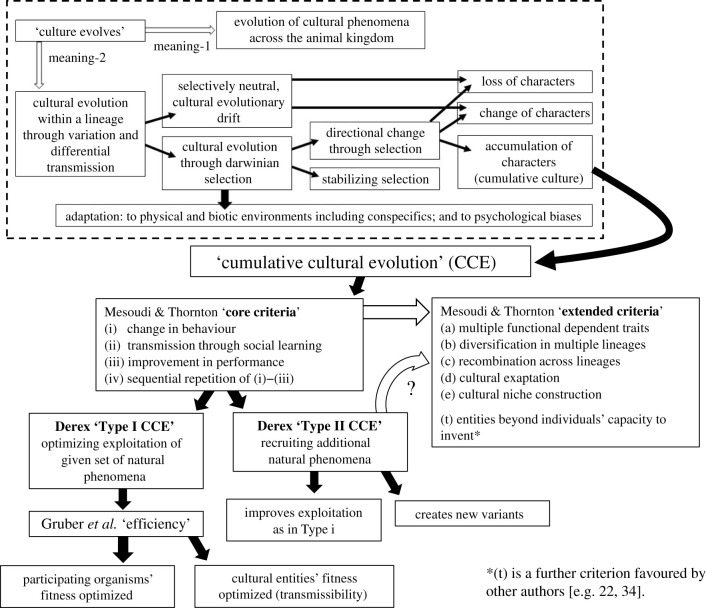
A taxonomy of distinctions between manifestations of ‘cultural evolution’. In the top, framed box (after [25]) are distinctions between two broad meanings of the assertion that ‘culture evolves’, followed by a dissection of forms of cultural evolution. The remainder of the figure illustrates distinctions within cumulative cultural evolution (CCE) due to [78,151,152], as discussed in the text. The white arrow between core and extended criteria for CCE indicates that the latter are potential variants within the category defined by the former. The white arrow between Type II CCE and the extended criteria indicates that whether the latter are causally dependent on the former is a remaining empirical question.

